# Tocilizumab for COVID-19 Acute Respiratory Distress Syndrome: Outcomes Assessment Using the WHO Ordinal Scale

**DOI:** 10.7759/cureus.12290

**Published:** 2020-12-26

**Authors:** Nosheen Nasir, Faisal Mahmood, Kiren Habib, Iffat Khanum, Bushra Jamil

**Affiliations:** 1 Internal Medicine, Aga Khan University, Karachi, PAK; 2 Internal Medicine, Aga Khan University Hospital, Karachi, PAK; 3 Medicine, Aga Khan University, Karachi, PAK; 4 Medicine/Infectious Diseases, Aga Khan University Hospital, Karachi, PAK

**Keywords:** ards, covid-19, cytokine release syndrome, il-6, tocilizumab

## Abstract

Introduction

Cytokine release syndrome in COVID-19 is characterized by hyperinflammation, which manifests as acute respiratory distress syndrome (ARDS), multiorgan failure, and high inflammatory parameters. Tocilizumab, an interleukin 6 (IL-6) antagonist has been used in COVID-19 ARDS with conflicting results from different parts of the world.

Objective

To study the treatment outcomes with tocilizumab in patients with COVID-19 ARDS and hyperinflammation using the World Health Organization (WHO) COVID-19 ordinal scale.

Methods

An observational study was conducted from Feb 2020 to May 2020 on COVID-19 ARDS patients with hyperinflammation.

Results

A total of 244 patients with COVID-19 were admitted, out of which 107 had ARDS. Thirty patients had both ARDS and hyperinflammation and received tocilizumab. The mean age was 62.5 years (SD: 13.5) and the majority were male (83%). The mean CRP pre-treatment was 217.5 mg/L and post 48 to 72 hours of tocilizumab treatment was 98.5 mg/L. Twenty-one patients (70%) also received concomitant intravenous (IV) methylprednisolone. Of the 30 patients, seven died and 20 recovered. Ten patients required intensive care unit admission and nine developed nosocomial infections. COVID-19-associated aspergillosis was diagnosed in three patients post tocilizumab treatment. Mortality was significantly higher in patients who developed a nosocomial infection and who required intermittent positive pressure ventilation (IPPV). Post-treatment, clinical improvement was observed in patients who had a median score of 5 on the WHO ordinal scale.

Conclusion

Our study supports the use of tocilizumab in COVID-19 ARDS patients with a pre-treatment median WHO ordinal severity score of 5 and recommends the monitoring of nosocomial infections and opportunistic infections.

## Introduction

COVID-19 was announced as a pandemic by the World Health Organization (WHO) in March 2020 and since then it has infected 5,113,706 people globally and resulted in 330,361 deaths as of date [1-2]. The life-threatening manifestation of COVID-19 is acute respiratory distress syndrome (ARDS) and is associated with significant morbidity and mortality [3]. The underlying pathogenesis of ARDS involves a dysregulated immune response leading to a cytokine release syndrome (CRS), referring to an excessive and uncontrolled release of pro-inflammatory cytokines. CRS in COVID-19 is characterized by hyperinflammation, which manifests as ARDS, multiorgan failure, and high inflammatory parameters [4]. Key in the development of the CRS is an exaggerated release of the proinflammatory cytokine Interleukin-6 (IL-6) and elevated IL-6 levels correlate with ARDS [5]. Marked elevation of C-reactive protein (CRP) (whose expression is propelled by IL-6) also serves as a biomarker to assess the severity of clinical CRS [3] and studies have used CRP and ferritin as surrogate markers of hyperinflammation [6-7].

Given the pivotal role of IL-6; it has been postulated that targeting IL-6 with available IL-6 inhibitors like tocilizumab may lead to clinical suppression of the CRS [8]. Data from clinical studies have been conflicting with regards to the efficacy of tocilizumab in COVID-19 ARDS. While limited studies from China have shown improved outcomes in COVID-19 patients with hyperinflammation and ARDS [9], a study from Italy did not show significant mortality benefit [10]. While randomized controlled trials are awaited, there is an urgency to explore therapeutic options that can help to avert ICU admissions, especially given the limited capacity in resource-constrained settings. Moreover, preliminary reports from the COVACTA trial (a study to evaluate the safety and efficacy of tocilizumab in patients with severe COVID-19 pneumonia) by the manufacturer have not shown a mortality benefit with the use of this drug in COVID-19 [11]. Hence, we would like to report our clinical experience of the management of ARDS and hyperinflammation with the IL-6 inhibitor tocilizumab in patients with critical COVID-19.

A preprint of this article (Treatment of ARDS and hyperinflammation in COVID-19 with IL-6 antagonist tocilizumab: a tertiary care experience from Pakistan) is available [12].

## Materials and methods

We conducted an observational study on COVID-19 patients with ARDS and hyperinflammation admitted to a 700-bedded tertiary care hospital. A cytokine storm or hyperinflammation was defined as either serum CRP of ≥100 mg/L or ferritin of ≥900 ng/mL or both [6]. ARDS was defined as per the WHO definition as having “onset within 1 week of a known clinical insult or new or worsening respiratory symptoms, chest imaging (radiograph, CT scan, or lung ultrasound) with bilateral opacities, not fully explained by volume overload, lobar or lung collapse, or nodules and respiratory failure not fully explained by cardiac and PaO2/Fio2 <300 mmHg” [13]. As per hospital COVID-19 management guidelines, patients were considered for treatment with intravenous tocilizumab if they fulfilled the above-mentioned criteria for ARDS and hyperinflammation and if they failed treatment with steroids in the first 24 to 48 hours, that is, if the patient’s partial pressure of oxygen (PaO2/fraction of inspired oxygen (FIO2) ratio decreased further AND/OR the patient’s oxygen requirement worsened or immediately if they fulfilled criteria for septic shock and required mechanical ventilation. Patients were not given intravenous (IV) tocilizumab if they had any contraindication, such as hepatitis, as indicated by transaminitis (alanine transaminase (ALT) of greater than three times the upper normal limit) and/or ongoing bacterial infection or tuberculosis. A single dose was administered intravenously to all patients who met eligibility and in those in whom there was no improvement in terms of a) oxygen requirement, b) increased chest infiltrates on chest radiograph, or c) decreased PaO2/FIO2 ratio, another intravenous dose was repeated at 12 hours. Demographics and clinical data from the hospital medical records were collected using a structured proforma. The outcomes assessed included in-hospital mortality, length of stay, and the development of nosocomial infection during hospitalization. In order to assess response to treatment, the WHO ordinal scale ranging from 1 to 8 where a score of one means no limitation of activities and 8 is death [14] was recorded at three time-points ( pre-tocilizumab, 48 hours post-tocilizumab, and then at five days post-tocilizumab or at last follow-up. The study was submitted for ethical approval to the AKUH ethical review committee and received exemption (ERC reference number: 2020-3650-10382). The data were anonymized, and no personal identifiers were recorded.

Categorical variables, such as gender and the development of nosocomial infection, were described as proportions, and continuous variables like age and length of hospital stay were described using mean, median, and interquartile range (IQR) values. Proportions for categorical variables as mentioned above were compared using the χ2 test or Fisher exact test where appropriate. Continuous variables were compared using the student's t-test or Wilcoxon rank-sum test as appropriate. Statistical analysis was performed using STATA ver 12 (STATA Corp., College Station, Texas). A p-value of less than 0.05 was considered statistically significant.

## Results

**Figure 1:**
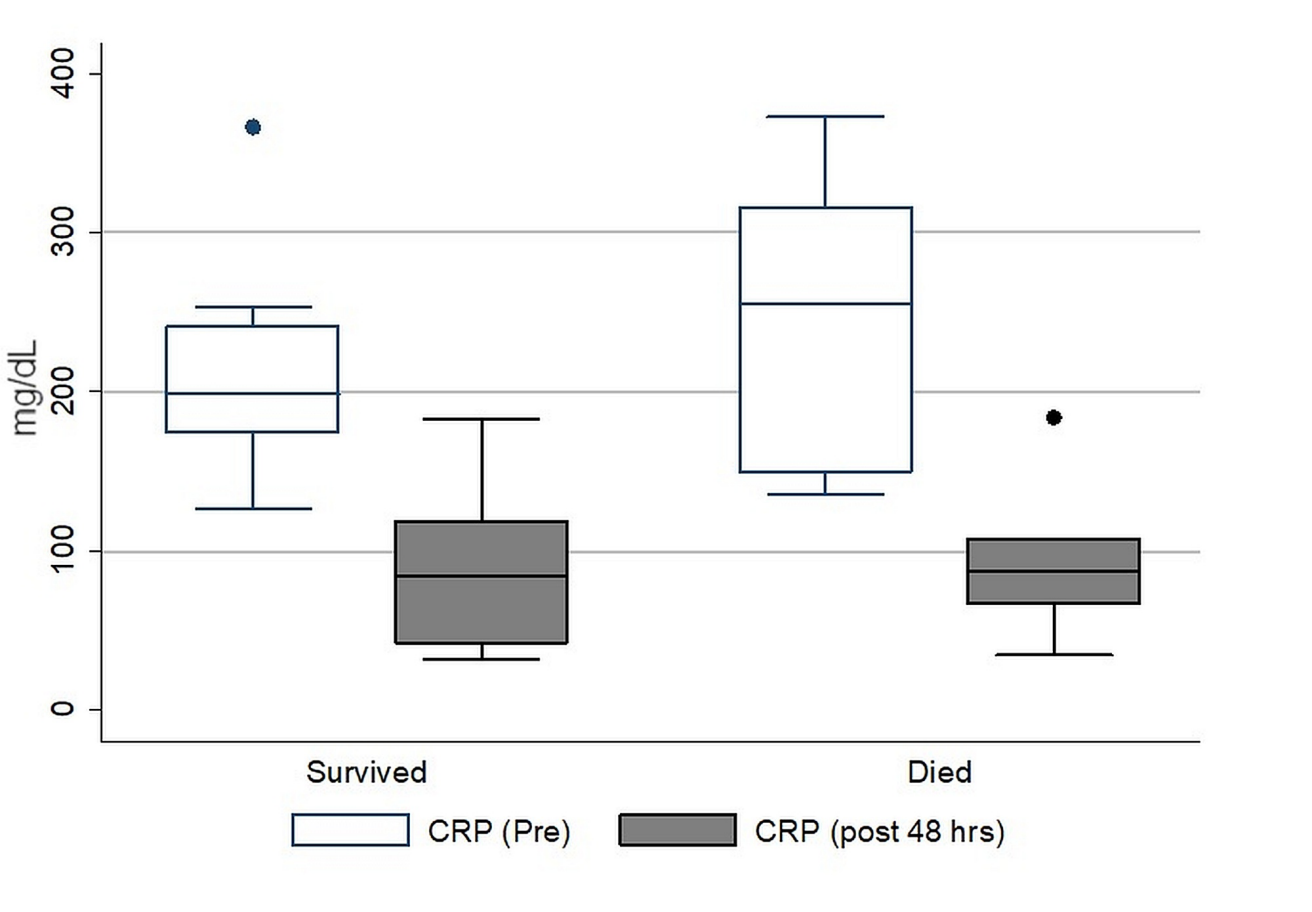
Comparison of C-reactive protein levels before and after the administration of intravenous tocilizumab in patients who died versus those who survived

A total of 244 patients with COVID-19 were admitted from February 26 to May 15, 2020, out of which 107 met WHO criteria for ARDS. Of these, there were 30 patients with ARDS who also met the criteria for hyperinflammation and qualified to receive tocilizumab. The clinical characteristics and outcomes of these 30 patients are summarized in Table 1. The mean age was 62.5 years (SD: 13.5 years), and the majority were male (83%). None of the patients had a rheumatological illness. The median dose of IV tocilizumab was 600 mg (range: 320 - 680 mg). No adverse effects were observed during or post-infusion. Twenty-one patients (70%) also received concomitant systemic steroids (IV methylprednisolone at a dose of 40 mg twice daily for three to five days). Of the 30 patients, seven died and 20 recovered while information was missing on three patients who left against medical advice. The mean length of hospitalization was 12 days (SD: 6.7). The mean CRP pre- and post-tocilizumab treatments in those who died compared to those who survived are shown in Figure 1.

**Table 1:** Clinical characteristics and outcomes of COVID-19 patients who received tocilizumab CRP: C-reactive protein

Demographics			
Number patients (N)	30		
Median age in years (IQR)	65 years (IQR: 55-72)		
Gender n(%)	Male	Female	
	25 (83%)	5 (17%)	
Length of stay mean ±SD days	12 ±6.7		
Pre-treatment ventilator status			
Assisted ventilation n(%)	IPPV	NIV	None
	10 (33%)	14 (48%)	6 (20%)
Drug management			
Tocilizumab dose median (range) mg	600 (320-680)		
Concomitant steroids n(%)	21 (70%)		
Outcome			
Acute-phase reactant response	Pre-tocilizumab	Post-tocilizumab	
CRP mean ± SD mg/L	217.5± 64.6	98.4 ±56.3	
Immunosuppression			
Nosocomial infection n(%)	9 (30%)		
Aspergillus infection/colonization n(%)	7 (24%)		
Final outcome	Dead	Alive	Unknown
n (%)	7 (23%)	20 (66%)	3 (10%)

**Figure 2:**
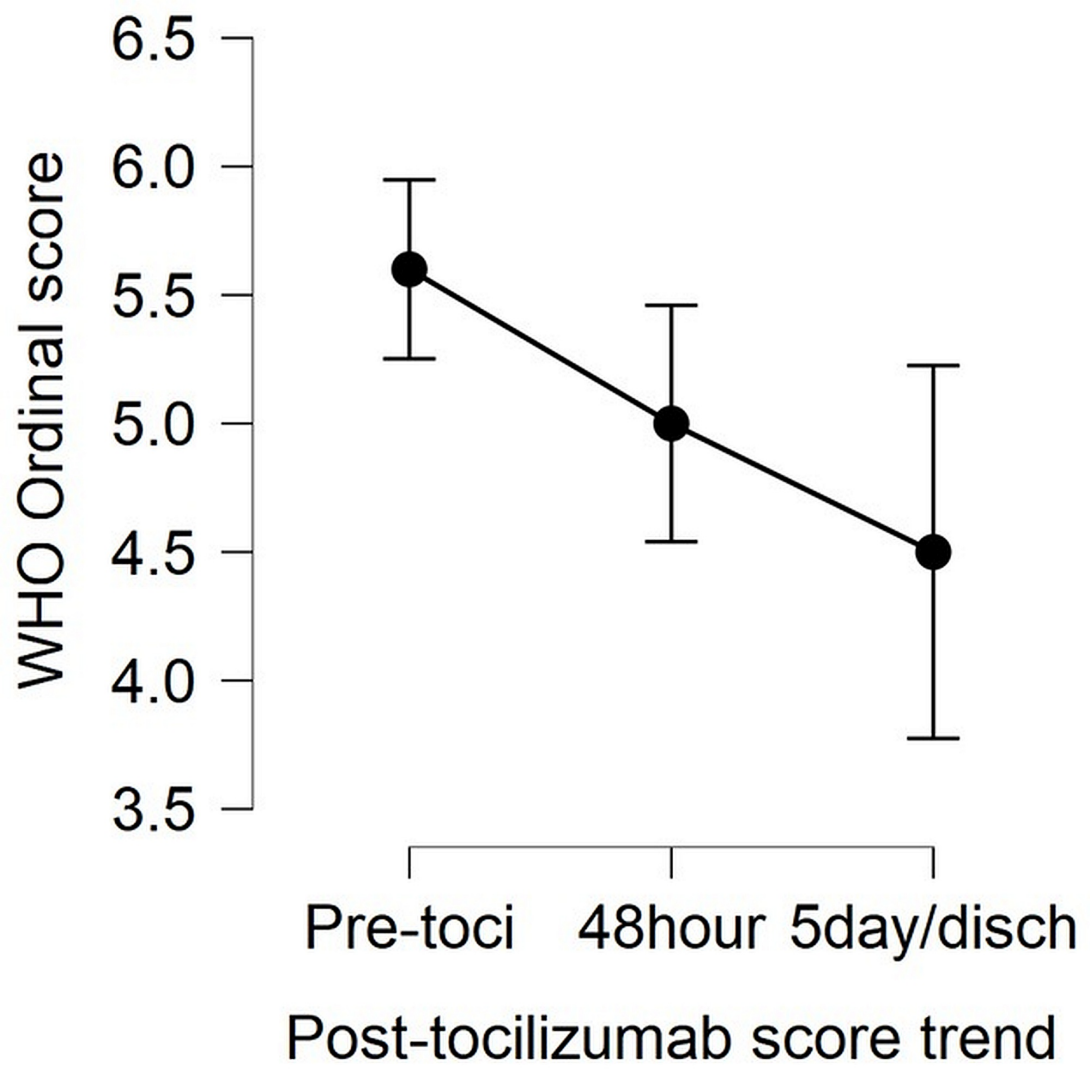
Comparison of median WHO ordinal severity scores for clinical improvement before and after the administration of intravenous tocilizumab in patients

Ten patients required intensive care unit (ICU) admission and intermittent positive pressure ventilation (IPPV), whereas 14 patients were managed on non-invasive ventilation (NIV). Nine patients developed nosocomial infections, of which six were hospital-acquired pneumonia (three with multi-drug resistant (MDR) acinetobacter, two with MDR Pseudomonas (P.) aeruginosa, and one with methicillin-resistant Staphylococcus aureus (MRSA). Patients who developed MRSA infection were treated with IV vancomycin and those with MDR acinetobacter or P. aeruginosa were treated with IV meropenem and colistin. Additionally, seven patients also isolated Aspergillus species from their respiratory specimens. Of these seven, only three patients were diagnosed with COVID-19-associated aspergillosis (based on clinical and radiological worsening and improvement in response to treatment) while in the rest, the aspergillus was considered a colonizer. Mortality was higher in patients who developed a nosocomial infection (p-value: 0.005) and who required IPPV (p-value: 0.023). The median WHO severity score before tocilizumab was 5 (range 4-7). While there was no change in the median WHO severity score 48 hours after tocilizumab, there was an overall improvement at five days (or last follow-up) with a median score of 3.5 (range 3 to 8) (Figure 2).

Out of 30 patients who received tocilizumab, 18 showed an improvement of at least one point on the ordinal score at 48 hours and 12 did not show any clinical improvement from their baseline pre-treatment score. None of the patients showed progression on the ordinal score at 48 hours. However, when pre-treatment scores were compared with Day 5/day of last follow-up score; five out of 30 patients who had a pre-treatment score of 7 had shown progression and died, five had shown no improvement and remained at the same score, whereas 20 had shown improvement from their baseline score of at least one point (p-value<0.001).

## Discussion

The WHO has raised an alarm over the numbers of cases that are exponentially increasing in the lower-middle-income countries (LMICs). It is a major cause of concern, particularly since the capacities of developed countries have been overwhelmed. Since the development of a vaccine is unlikely in the near future, the focus has been on treatment and the compassionate use of certain medications. Tocilizumab is a monoclonal antibody targeting the receptor of IL-6; a pro-inflammatory cytokine involved in the pathogenesis of ARDS seen with COVID-19 [8]. Data are urgently needed from developing countries, as a “one-size fits all” strategy cannot be used in resource-constrained regions where healthcare capacities are already overstretched [15]. Moreover, whether tocilizumab is a cost-effective option in developing countries also requires exploration because of the differences in case fatality rates from the different parts of the world suggesting that the same approaches may not be regionally relevant. We conducted an observational study describing patient outcomes in those critically ill patients of COVID-19 who received tocilizumab intravenously for hyperinflammation and ARDS. Most of the data regarding its off-label use in COVID-19 has been from studies conducted in China and parts of Europe (Table 2). The age and gender distribution of our patient population were similar to those reported in studies from China and Italy [10,16]. The mortality data has been conflicting from Italy with Colaneri et al. reporting no benefit [10], whereas Sciascia et al. showed reduced mortality in those who received treatment with tocilizumab [17]. On the other hand, outcomes have been consistently reported to be good in studies from China. At the time of submission, some had not been peer-reviewed and others did not take into account concomitant treatments [9,16]. Our study has reported a mortality of 23%, which is similar to other studies, but we also report the concomitant use of systemic steroids in 70% of the patients, which may have contributed to immunomodulation. None of the studies so far have reported nosocomial infection, Aspergillus infection, or colonization. Since IL-6 antagonism can potentially predispose to worse outcomes in infections, this is an important observation in our study and can have implications in developing countries where there is a higher incidence of MDR infections. Our study also found clinical improvement in patients who had a pre-treatment score of 5 on the WHO ordinal scale, i.e., hospitalized with non-invasive ventilation or oxygen support, and none to minimal improvement in patients who had pre-treatment of 7, i.e. on ventilation and additional organ support, including vasopressors and renal replacement therapy. Ruiz et al. reported improvement post-tocilizumab using the ordinal scale over a longer follow-up period of 14 days in a single-center observational study [18]. Another study showed a better outcome in patients requiring mechanical ventilation with the use of tocilizumab, which is in contrast to our findings [19]. On the other hand, preliminary findings from the COVACTA trial have not shown promising results but have been criticized, as they included patients with greater severity of illness despite data from observational studies suggesting better outcomes in earlier disease and outcome assessment at one specified day, which may not account for individual differences in patients and concomitant treatment-related information, particularly systemic steroids, was not sufficient [11]. Although our study is limited because of the small sample size, lack of a comparator group, and single-center design, this is expected given the availability and cost of tocilizumab in a developing country, which makes it imperative to understand its potential for use in our setting. All the studies exploring outcomes with tocilizumab are limited due to their small sample sizes and observational design highlighting the need for randomized controlled trials. However, given the rapidity of the spread of COVID-19 infection, real-time data is needed, particularly from LMICs, which are experiencing an exponential increase in the number of cases. Our study has taken into account the fact that the peak effect of tocilizumab may be prolonged and earlier outcomes have more relevance in view of the acuteness of presentation and rapid progression in the absence of immunomodulation in patients with hyperinflammation and ARDS. Hence, the appropriate timing of tocilizumab is perhaps a key factor for improving survival [20].

**Table 2:** Summary of studies describing experience with tocilizumab use in severe COVID-19 infections

Study	Country of study	Number of patients who received IV TOCILIZUMAB	Study design	Outcomes	Conclusion
Luo P et al. [16]	China	15	Observational	3/5 (20%) mortality, 2/15 with disease aggravation	Good response in patients with tocilizumab
Colaneri M et al. [10]	Italy	21	Observational	5/21 (24%) Day-7 mortality on Day 7. No difference compared with those who didn’t get tocilizumab	Tocilizumab administration did not reduce ICU admission and mortality rate
Sciascia S et al. [17]	Italy	34	Pilot prospective open single-arm	4/31 (12%) mortality in the tocilizumab arm	May benefit in severe disease
Xu X et al. [21]	China	21	Observational	No mortality	Good outcome
Van Kraaij et al. [22]	Netherlands	1	Case report	Survived	Good outcome
Klopfenstein T et al. [23]	France	20	Case-control	25% mortality in the tocilizumab group versus 48% in the no tocilizumab group	Good outcome

## Conclusions

In conclusion, our study supports the use of tocilizumab in COVID-19 ARDS patients with a pre-treatment median WHO ordinal severity score of 5 (on oxygen support or NIV), whereas they may not benefit in terms of clinical outcomes if it is administered at a score of 7 (on mechanical ventilation and organ support). We also recommend the monitoring of patients who receive the medication for nosocomial and opportunistic infections, which may lead to adverse outcomes in COVID-19 patients. Furthermore, in view of issues with the COVACTA trial; a multicountry randomized controlled pragmatic trial is needed, which takes into account the time factor in outcome assessment and differences in standard of care practiced in different regions in the setting of the pandemic.
